# Anesthesia for Patients With Liver Disease

**DOI:** 10.5812/hepatmon.19881

**Published:** 2014-07-01

**Authors:** Poupak Rahimzadeh, Saeid Safari, Seyed Hamid Reza Faiz, Seyed Moayed Alavian

**Affiliations:** 1 Department of Anesthesiology and Pain Medicine, Rasoul Akram Medical Center, Iran University of Medical Sciences, Tehran, IR Iran; 2 Middle East Liver Disease Center (MELD), Tehran, IR Iran; 3 Baqiyatallah Research Center for Gastroenterology and Liver Diseases, Baqiyatallah University of Medical Sciences, Tehran, IR Iran

**Keywords:** Liver Disease, Anesthesia, Regional Anesthesia, Epidural Anesthesia, Spinal Anesthesia

## Abstract

**Context:**

Liver plays an important role in metabolism and physiological homeostasis in the body. This organ is unique in its structure and physiology. So it is necessary for an anesthesiologist to be familiar with various hepatic pathophysiologic conditions and consequences of liver dysfunction.

**Evidence Acquisition:**

We searched MEDLINE (Pub Med, OVID, MD Consult), SCOPUS and the Cochrane database for the following keywords: liver disease, anesthesia and liver disease, regional anesthesia in liver disease, epidural anesthesia in liver disease and spinal anesthesia in liver disease, for the period of 1966 to 2013.

**Results:**

Although different anesthetic regimens are available in modern anesthesia world, but anesthetizing the patients with liver disease is still really tough. Spinal or epidural anesthetic effects on hepatic blood flow and function is not clearly investigated, considering both the anesthetic drug-induced changes and outcomes. Regional anesthesia might be used in patients with advanced liver disease. In these cases lower drug dosages are used, considering the fact that locally administered drugs have less systemic effects. In case of general anesthesia it seems that using inhalation agents (Isoflurane, Desflurane or Sevoflurane), alone or in combination with small doses of fentanyl can be considered as a reasonable regimen. When administering drugs, anesthetist must realize and consider the substantially changed pharmacokinetics of some other anesthetic drugs.

**Conclusions:**

Despite the fact that anesthesia in chronic liver disease is a scary and pretty challenging condition for every anesthesiologist, this hazard could be diminished by meticulous attention on optimizing the patient’s condition preoperatively and choosing appropriate anesthetic regimen and drugs in this setting. Although there are paucity of statistics and investigations in this specific group of patients but these little data show that with careful monitoring and considering the above mentioned rules a safe anesthesia could be achievable in these patients.

## 1. Context

The liver plays an important role in homeostasis of many Physiological systems, such as food and drug metabolism, plasma protein synthesis, critical hemostatic factors, detoxification and exclusion of many endogenous and exogenous substances (1, 2). On the other hand, it is involved in host immune reactions to injury, sepsis, and inflammation (3-5).

The liver receives 25% of cardiac output as a result of having dual afferent blood supply. About 70% of hepatic blood flow is supplied by portal vein, and the rest by hepatic artery. Under normal conditions, each blood vessel contributes in supplying roughly 50% of liver’s oxygen. Portal vein flow is not regulated and is only affected by systemic hypotension and decreases in cardiac output (1, 2).

Anesthesia in patients with hepatic disease is a pretty challenging condition even for the expert anesthetist.

## 2. Evidence Acquisition

We searched MEDLINE (Pub Med, OVID, MD Consult), SCOPUS and the Cochrane database for the following keywords: liver disease, anesthesia and liver disease, regional anesthesia in liver disease, epidural anesthesia in liver disease and spinal anesthesia in liver disease, for the period of 1966 to 2013. In addition, we examined cited references in these studies with the same keywords again. Abstracts or unpublished studies were excluded from the study. All randomized clinical trials, case series and case report studies with the above mentioned contents were included in review process. In the field of regional anesthesia and hepatic disease, there was lack of proper previous study in the literature. So in order to expand the research field we tried to have a look on liver anesthesia. Totally, 66 articles were eligible and enrolled in this study.

## 3. Results

### 3.1. Regulation of Hepatic Blood Flow

Studies have shown that up to a 50% decline in portal flow is modulated by maintaining hepatic artery tone to keep perfusion to the liver. This is firstly mediated via the hepatic arterial buffer response, which mutually varies hepatic arterial blood flow to changes in portal flow, because of adenosine. The response is excited by low pH and O_2_ content and increased PCO_2_. Some factors such as using volatile anesthetics and Cirrhosis of liver debilitate this mutual relationship and render the liver vulnerable to ischemia (6, 7).

Anatomically speaking, this major organ has complex innervations and perfusion. Perfusion condition was discussed earlier and innervations of the liver is by two main pathways which are as follows:

1. Anterior plexus surrounding hepatic artery which includes postganglionic sympathetic fibers from celiac ganglia and parasympathetic fibers from anterior Vagus nerve.
2. Posterior plexus surrounding portal vein and bile duct which includes postganglionic sympathetic fibers from right Celiac ganglia and parasympathetic fibers from posterior Vagus nerve.

Studies have shown that when sympathetic nerve fibers dominate, it causes an increase in vascular resistance and decrease in blood volume. Excitation also increases glycogenolysis and gluconeogenesis profile, but Parasympathetic stimulation increases glucose uptake and glycogen synthesis (8-10). So the autonomic nervous system plays an important role in anesthetic management of these patients.

Acute or chronic liver dysfunction may destruct the body response to anesthesia and surgery in some important ways and cause new reactions. Specific anesthetic and hemodynamic abnormalities can create serious changes and consequences on postoperative liver function. Recently, clinical medicine has responded to public expectations and achieved scientific progress with great developments in caring for the patients with liver disease. Nowadays, fear of severe hepatic disease has been decreased. Even patients with end-stage liver undergo surgery during the last two years of their life. In addition to lower risk drugs, less invasive surgical techniques have made surgery possible for patients in extreme conditions (11). there is a general understanding among anesthesiology and intensive care physicians that patients with liver disease are at sensible risk when undergoing anesthesia and surgery (12-18). Gastroenterologists and hepatologists are usually asked to evaluate patients with liver disease pre-operatively in order to optimize the condition prior to surgery. Numerous and diverse surgical procedures may be performed for these patients so a variety of anesthetic techniques could be used depending on the type of surgery.

Although different anesthetic regimens are available in modern anesthesia world, but anesthetizing these patients is still really tough, and decision making quite challenging. Spinal or epidural anesthetic effects on hepatic blood flow and function is not clearly investigated, considering both the anesthetic drug-induced changes and outcomes. Surgical stress, especially in laparotomy surgeries of patients with liver disease is associated with high mortality. In some special situations mortality rate reaches up to 85% to 95% (19).

There are some related risk factors for surgical morbidity and mortality which include, male gender, presence of Ascites, Cirrhosis diagnosis, high creatinine concentration, chronic obstructive pulmonary disease, postoperative infection, upper gastrointestinal bleeding, intraoperative hypotension and some other factors (20). Clinically, the anesthesiologist may divide patients with liver disease into two major groups:

1. Parenchymal liver disease, such as acute and chronic viral hepatitis, Cirrhosis of liver (with or without high portal hypertension) and some other disorders.
2. Patients with cholestasis, such as obstruction of extra hepatic bile ducts.

In the first group, an increase in aminotransferas enzymes occurs. Actually, parenchymal liver disease is a hyperdynamic condition in body, which is usually associated with reduction in vascular resistance, peripheral vasodilatation, increased arterio-venous shunting, increment in circulatory blood volume and cardiac output. Also, there are chances of cardiomyopathy, decreasing the difference in arterio-venous oxygen content and lowering portal blood flow in these patients. It should be noted that in severe liver insufficiency, due to right shift in oxygen-hemoglobin curve, pulmonary shunts and Ascites induced hypoventilation, hypoxemia may occur. However, there are other co-existing problems accompanying hepatic disease in these patients which are as follows: anemia, leucopenia, thrombocytopenia and coagulopathy. Encephalopathy, renal dysfunction, including hepatorenal syndrome, and ascites are also common in these patients.

When anesthesia induction of patients with liver disease is done, oxygen supply-demand relationship should be considered. The major target is to maintain adequate pulmonary ventilation and cardiovascular function. For this reason, cardiac output, blood volume, and perfusion pressures should be kept in the normal range. Arterial hypotension should always be avoided. Arterial hypotension may be drug induced or due to inadequate blood volume replacement or even overdose of inhalational anesthetics. Investigations have shown that the outcomes of these effects are vasodilation and a reduction in perfusion pressure, plus a decline in blood velocity. These can lead to improvement in oxygen extraction in all tissues, including the preportal area.

The final result is a decline in portal oxygen content which can lead to compensatory increase in hepatic arterial blood flow. Unfortunately, in severe hepatic disease, these compensatory mechanisms do not work well or have been destroyed (21-24).

### 3.2. Difficulties on Assessment of Preoperative Risk

Significant functional reserve and nonspecific nature of liver blood tests, cause difficulty for evaluating the extent of liver dysfunction. This prevents proper assessment of preoperative risk. In addition, there is lack of good retrospective studies and case series. Limited articles were found on the risk of anesthesia in non-cirrhotic patients. Those with asymptomatic biochemical abnormalities and minor liver dysfunction would generally tolerate the surgery well, and it is not recommended to over check them before procedure. Nevertheless, it is always difficult to be certain, because abnormal transaminase could result in significant morbidity or mortality after surgery. Patients with decompensatory cirrhosis are at great risk. So extreme care should be taken when anesthetizing them. In such conditions, the cost and benefit of surgery must be carefully weighed. If surgery seems necessary, the patient’s condition will need to be optimized prior to operation.

Liver function should be kept and is crucial to maintain homeostasis in the preoperative period and in critical illness. Preoperatively, however, liver function is impaired and hepatocellular damage occurs. Although maintaining liver function is always necessary, this function would be impaired during surgery.

Friedman has offered a list of patients who should not undergo elective surgery. These include, patients with acute viral or alcoholic hepatitis, fulminant hepatic failure, severe chronic hepatitis, Child’s class C cirrhosis, severe coagulopathy, severe extra hepatic complications including hypoxemia, cardiomyopathy, or acute renal failure. In major surgeries with critical illness, hepatic dysfunction is related to poor prognosis. In a mixed intensive care unit patient population, hepatic dysfunction soon after admission, increased mortality rate by 80% (22-26).

### 3.3. Principles of Anesthetic Management in Hepatic Patients

It is generally accepted that risk of surgery cannot be isolated from risk of anesthesia. Inhalation anesthetics, narcotics, and intravenous sedative-hypnotic agents are generally well tolerated in patients with compensatory liver disease. They should be used with caution in patients with decompensatory hepatic dysfunction, because they may cause prolonged effects on consciousness, hemodynamic and result in hepatic Encephalopathy.

Studies showed that in healthy volunteers, hepatic blood flow decreases by 35% to 42% in the first 30 minutes of anesthesia induction. In patients with hepatic dysfunction, especially cirrhosis cases, compensation for reduced portal blood flow does not occur under anesthesia. This may cause more hepatic dysfunction, difficulty in anesthesia management and postoperative loss of consciousness (23-27).

Whenever possible, regional anesthesia might be used in patients with advanced liver disease. In these cases lower drug dosages are used, considering the fact that locally administered drugs have less systemic effects. This lowers the possibility of loss of consciousness and delayed recovery due to difficulty in drug metabolism. Both neuroaxial and regional anesthesia could be considered in patients with hepatic failure. Total consumption dose while performing regional anesthesia should be cautiously calculated and close monitoring for any possible side effects is necessary. Coagulopathy should be considered as a contraindication to some types of regional anesthesia. Regional techniques can be considered in selected patients with acceptable coagulation profile. Regional anesthesia attenuates surgery-induced stress responses. these include, increase in levels of corticosteroid hormone and catecholamine. Regardless of the effects of stress hormones on hemodynamic and circulation, they are thought to play an important role in depressing immune function (10, 28-31). In all cases under anesthesia, arterial blood pressure should be preserved and sympathetic stimulation avoided.

In abdominal or thoracic surgeries, thoracic epidural anesthesia (TEA) induces excellent pain relief and may reduce postoperative mortality. Also in lower abdominal and limb surgeries, lumbar epidural anesthesia is helpful for anesthetic management and postoperative care. Both techniques have been introduced as multimodal analgesia for major surgery (32-38).

Previous studies have shown that sympathetic nerve activity plays a crucial role in hepatic injury. Immune responses and stressful events induce liver injury in laboratory cases (39). In animal studies, autonomic denervation of the liver reduced hepatic injury. This finding indicated the important action of sympathetic activity (40, 41). In sepsis, adrenoreceptors influence hepatocellular dysfunction and immune responses (42, 43). Sympathetic activity also affects regeneration after liver resections (44). It is speculated that the key mechanism of protective and supportive effects of epidural anesthesia is sympathetic block (45, 46). Intestinal effects of TEA have been extensively investigated in clinical and animal studies (47-49). In contrast, the knowledge about hepatic effects of TEA is limited (50). The influence of thoracic or lumbar epidural anesthesia on hepatic microcirculation has not been investigated yet.

Due to unknown mechanism of TEA effects on hepatic microvascular injury and leukocyte adhesion in critical illness, an animal study was performed to test the following hypothesis: The influences of TEA on hepatic microvascular perfusion and leukocyte activation in healthy cases. Reduction effect of TEA on hepatic microvascular disturbance, inflammation, and apoptosis in critical illness induced by severe acute pancreatitis (51).

The results showed that, in both presinusoidal and postsinusoidal sphincters, sympathetic and parasympathetic regulation of liver blood flow occurs. Under resting conditions in healthy cases, there is little tonic sympathetic activity, whereas vagal nerve activity tonically influences hepatic blood flow. Hepatic denervation did not change resting blood flow in animals. This only impaired hepatic buffer response during reduced portal inflow, which is a helpful response (52). In contrast to resting condition, in the face of increased sympathetic tone, hepatic microcirculation and cell injury are significantly affected. In healthy rats, electrical stimulation of the hepatic sympathetic nerves induced a strong decrease in hepatic blood flow (53). Stimulants of sympathetic activity such as inducing psychic stress in adult male mice, baroreceptor response, acute urinary retention, or inserting painful stimuli during anesthesia reduce regional hepatic blood flow (54, 55). In animal models of liver surgery and manipulation, hepatic denervation exerted differential effects on living compared to brain-dead animal models, which could possibly be related to altered sympathetic activity (56).

It is assumed that sympathetic block by epidural anesthesia might have mediated the decreased vasoconstrictive response in severe acute pancreatitis. No such response was recorded in healthy liver models (51). There are no remarkable studies regarding regional anesthesia efficacy in patients with liver disease. This is a new field which needs to be investigated and practiced more in the future.

Considering general anesthesia in these patients, investigations showed that among the inhalation anesthetics, Halothane should be avoided because maintaining hepatic blood flow is critical in hepatic patients. This anesthetic agent leads to the most prominent decrease in hepatic blood flow, oxygen supply and postoperative hepatic dysfunction of all inhalation anesthetics. In addition, immunologically mediated severe postoperative halothane hepatitis may be followed by Halothane anesthesia. Isoflurane seems to be a better choice if an inhalational technique is selected in these patients (57-62). Newer volatile anesthetics such as Sevoflurane and Desflurane, have not been studied as much as Halothane and Isoflurane. A few indirect comparisons of Sevoflurane and Desflurane with Isoflurane and Halothane suggest that, although there is no significant difference between them but Sevoflurane could have some advantages over other volatile anesthetics (63-67). Further studies are required to make definite conclusions and selections about these anesthetic agents. Nitrous Oxide has been used in patients with advanced hepatic disease for many years without any complication. Some authors believe that using Nitrous Oxide in patients with advanced liver disease, may jeopardize oxygenation as a result of its sympathomimethic effects. On the other hand, long anesthesia with Nitrous Oxide might result in accumulation of gas in the intestinal lumen and subsequent intestinal distension.

Opioids have been used successfully in patients with hepatic disease. However, certain pharmacological consequences such as delayed drug clearance and prolonged half-life should be considered. Fentanyl is considered the opioid of choice in these patients because when used in relatively moderate doses, it does not decrease hepatic oxygen and blood supply, nor does it prevent increases in hepatic oxygen requirements (68, 69).

Spasm induction of Oddi sphincter was found by opioid usage with 3% incidence rate. Atropine, Naloxane, Glucagon, Nitroglycerin, volatile anesthetics, and other drugs can treat this spasm. Considering all the above mentioned anesthetic drugs and all the formerly written advices regarding anesthesia induction in hepatic patients, one should keep in mind that, the choice of anesthetic management should follow these rules: keep adequate pulmonary ventilation, cardiac output, and arterial pressure. While reviewing previously described anesthetic drugs it seems that anesthetic management using inhalation agents (Isoflurane, Desflurane or Sevoflurane), alone or in combination with small doses of fentanyl can be considered as a reasonable regimen. When administering drugs, anesthetist must realize and consider the substantially changed pharmacokinetics of some other anesthetic drugs. For instance, in patients with liver disease half-life of lidocaine and Benzodiazepines may increase by more than 300% and 100% respectively. Drugs ,such as Sodium Pentothal, with high affinity to albumin have a decline in volume of distribution. Therefore, dose of these drugs should be reduced. Among intravenous anesthetic agents, Propofol is the anesthetic drug of choice in patients with liver disease. It has short half-life even in patients with decompensated Cirrhosis. However, for many drugs, due to edema or increase in Gamma Globulin, the volume of distribution can be significantly increased, causing a necessity to increase the first effective dose of the drug.

As a general rule, any long acting narcotics and sedatives should be avoided in Cirrhotic patients. Narcotics like Fentanyl, Sufentanil and sedatives like Oxazepam, Lorazepam, in conjunction with some volatile anesthetics like Sevoflurane or intravenous anesthetics like Propofol are recommended (53, 68-72).

For muscle relaxants please keep in mind that clearance of drugs such as d-tubocurarine and Pancuronium because of decline in hepatic blood flow and hepatic metabolic and excretory functions, as well as impaired renal function, have decreased and therefore the effect can be prolonged. Studies showed that advanced hepatic disease does not significantly affect the pharmacokinetics of Vecuronium. Atracurium has a theoretical advantage because its metabolism is not dependent to hepatic function. So, clearance and elimination half-life of Atracurium in patients with impaired hepatic or renal function is not particularly different from those who have normal hepatorenal function. However, it has been found that because of larger volume of distribution, distribution half-lives are shorter in patients with severe hepatorenal dysfunction compared to normal individuals. Careful injection of any muscle relaxant according to transcutaneous nerve stimulator monitoring is beneficial. The best way to avoid complications is to titrate drugs against effects. Ultimately, in addition to logical selection of anesthetic drugs, close monitoring of all patients in this group is mandatory. It is the key factor of safe and harmless anesthesia.

However, judicious selection of anesthetic type management (either general, regional, or monitored anesthesia care), did not have favorable impact on mortality in some reported studies(60, 61). It is generally accepted that induction of a safe anesthesia in this specific group needs special attention, care, drugs and constant careful monitoring pre-operatively (13, 72, 73).

### 3.4. Coagulopathy

Coagulation management in this specific population is not quite different from other groups. For management of coagulopathy and surgical blood loss, like any other patient, these groups could be treated by administration of red blood cells, fresh frozen plasma, Platelets, and cryoprecipitate. In order to perform pharmacological treatment of hepatic-associated coagulopathy, the following drugs could be considered: Aminocaproic acid, Tranexamic acid, conjugated Estrogen, and activated recombinant factor VII. Thromboelastography may be helpful in identifying the cause of coagulopathy and can guide administration of coagulation products.

## 4. Conclusions

Despite the fact that anesthesia in chronic liver disease is a scary and pretty challenging condition for every anesthesiologist, this hazard could be diminished by meticulous attention on optimizing the patient’s condition preoperatively and choosing appropriate anesthetic regimen and drugs in this setting. Although there are paucity of statistics and investigations in this specific group of patients especially in performing regional anesthesia in these patients, but these little data show that with careful monitoring and considering the above mentioned rules a safe anesthesia could be achievable in these patients.
